# Biology of melanocytes in mammals

**DOI:** 10.3389/fcell.2023.1309557

**Published:** 2023-11-22

**Authors:** Ying-Zhe Cui, Xiao-Yong Man

**Affiliations:** Department of Dermatology, Second Affiliated Hospital, Zhejiang University School of Medicine, Hangzhou, Zhejiang, China

**Keywords:** melanocytes, pigmentation, melanin synthesis, melanocyte stem cells, melanoblasts, neural crest cells, Wnt signaling, epidermal keratinocytes

## Abstract

Melanocytes, which originate from the neuroectoderm, are specialized cells responsible for producing pigments and possessing a dendritic morphology. These cells migrate to the epidermis and follicles, contributing to skin and hair pigmentation during embryonic development. The remarkable self-renewal capacity of melanocytes enables them to effectively restore hair and skin pigmentation. The synthesis of melanin to safeguard the skin against damage caused by ultraviolet radiation, as well as the enigmatic immune function of melanocytes, demonstrate their indispensable contributions to maintaining cutaneous homeostasis. The regulation of cutaneous pigmentation involves an intricate network influenced by intrinsic cellular signals within melanocytes and extracellular cues. Therefore, this paper provides a comprehensive review of the role of melanocytes in skin biology. This in-depth analysis could open novel avenues for research aimed at the prevention and treatment of skin disorders.

## 1 Introduction

Melanocytes, referred to as pigment cells, are abundant within the human epidermis, with an approximate of 1,500 cells per square millimetre (Kanitakis, 2002). These pigmented cells are generally located in the dermis or epidermis and play a crucial role in skin pigmentation. They migrate along the dorsolateral pathway to connect the dermomyotome and the overlying ectoderm (Petit and Larue, 2016).

Melanoblasts, the precursors of melanocyte, can generate melanocyte stem cells (MSCs) and melanocytes during mouse embryo development (Cable et al., 1995; Mackenzie et al., 1997; Nishimura et al., 2002). Melanocytes are typically identified by the expression of melanocyte-specific proteins, including dopachrome tautomerase (DCT), tyrosinase-related protein 1 (TYRP1), tyrosinase (TYR), Pmel17/gp100, melanoma antigen recognized by T cells 1 (MART1), and/or microphthalmia-associated transcription factor (MITF) (Belote et al., 2021). However, identifying MSCs is more challenging because of their lack of melanin production. In addition, MSCs do not exhibit the typical protein markers (Nishimura et al., 2002). DCT and/or v-kit Hardy-Zuckerman 4 feline sarcoma viral oncogene homolog, also known as CD117 (C-KIT) may serve as detectable markers for these cells (Sun et al., 2019; Infarinato et al., 2020).

Technological advancements have gradually unveiled the specific functions and locations of melanocytes. Melanocytes and melanin have been detected in different anatomical regions, including the stria vascularis of the cochlea, the substantia nigra, leptomeninges, locus coeruleus within the brain, cardiac tissue, and adipose tissue (Plonka et al., 2009; van Beelen et al., 2020; Hutcheson et al., 2021; Ikeda et al., 2021). Moreover, Schwann cell precursors associated with nerves contribute to melanocytes in extracutaneous locations such as the heart, inner ear, supraorbital regions, and brain (Kaucka et al., 2021). Cells similar to melanocytes in the heart and pulmonary veins trigger atrial arrhythmia, whereas melanocytes with characteristics resembling macrophages residing adjacent to vessels in the inner ear maintain the integrity of the barrier between the intrastrial fluid and blood (Levin et al., 2009; Zhang et al., 2012). Melanocytes have also been identified in the limbal region, where they maintain limbal epithelial stem cells, regulate immune responses, and promote angiostasis (Polisetti et al., 2021).

This review provides a comprehensive summary of the developmental origins of melanocytes in mammals and elucidates how they are maintained by MSCs. We also explored the molecular mechanisms underlying melanocyte migration. This paper includes recent findings from studies that provide insights into the molecular mechanisms that influence skin and hair pigmentation in mammals. Furthermore, this review discusses the immune functions of melanocytes and outlines future research directions in the field of melanocyte biology.

## 2 The origin of melanocytes

**FIGURE 1:**
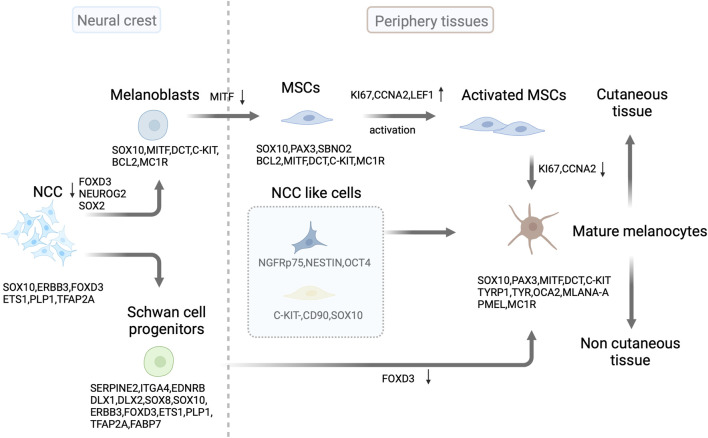
Source and development of melanocytes. In mammals, melanoblast lineages are derived from neural crest cells (NCC) through the downregulation of FOXD3 and NEUROG2/or SOX2 in progenitor cells. SOX10 expression continues in the melanoblast lineages, followed by MITF, DCT, and C-KIT expression. After colonizing developing embryonic hair follicles, some melanoblasts differentiate into melanocytes and produce the pigment (melanin) that colors the first hair cycle. Some melanoblasts form melanocyte stem cells (MSCs) in the hair follicle bulge and secondary hair germ cells by downregulating MITF expression. These stem cells replenish mature melanocytes through activation and subsequent proliferation of transit-amplifying cells in the hair cycle. Schwann cell progenitors derived from SOX10-expressing NCC contribute to the population of pigmented cells by downregulating FOXD3 expression. In the skin, NCC like progenitor cells can differentiate into mature melanocytes. This illustration was created using BioRender.com.

During the initial migration, a sequence of shared transcriptional states is observed during the progression of neural crest cells (NCCs), followed by branching pathways that determine their fate. NCCs express pan-neural crest marker genes, including *Sox10*, *Erbb3*, *Foxd3*, *Ets1*, *Plp1*, and *Tfap2a* (Kastriti et al., 2022). These cells undergo differentiation via characteristic lineage-restriction events involving the simultaneous expression and rivalry of genes that influence alternative destinations. Furthermore, specification of neural and melanocyte cell types may occur through the sequential expression of lineage-specific transcription factors (Soldatov et al., 2019). During migration to the intracranial space, melanocyte-destined cells stall in the migration staging area (MSA), where they express the microphthalmia-associated transcription factor (MITF) (Weston, 1991; Nakayama et al., 1998). Neurogenin 2 (Neurog2), the transcription factor required for sensory neurogenesis, is expressed in NCCs between Embryonic Day (E) 8.5 and E10.5, when they delaminate from the neural tube (Sommer et al., 1996). Neurog2 is transiently expressed in all neural crest derivatives during differentiation. It has two expression peaks: one early after NCC delamination, which has minimal direct regulatory impact; and another late after the commencement of sensory neurogenesis, which can be connected to the corresponding regulatory activity. Neurog2 knockout from E9.5–E15.5 promotes skin melanocyte generation. Thus, Neurog2 is involved in the early repression of melanocyte specification (Soldatov et al., 2019) (Figure 1). In contrast, MITF expression only begins at E10.5, which encourages melanocyte development. There was low expression of Neurog2 at E11.5 within the MSA, whereas MITF was highly expressed in NCCs (Hari et al., 2012). However, the relationship between MITF and Neurog2 remains unclear.

Hair follicle (HF) MSCs located in the hair bulge and secondary hair germ (sHG) are the main sources of melanocytes. MSCs are immature, slow-cycling, and self-sustaining cells that can renew melanocytes within hair follicles (Nishimura et al., 2002; Sun et al., 2023). They exhibit specific molecular expression patterns that are positive for DCT and paired box 3 (PAX3) and negative for TYR, TYRP1, MITF, lymphoid enhancer-binding factor-1 (LEF1), SRY-Box transcription factor 10 (SOX10), and MKI67. In contrast, transiently amplified (TA) and terminally differentiated cells express these markers (Osawa et al., 2005). Additionally, C-KIT expression can be used to identify MSCs in hair follicles. A C-KIT-CreER-driven model and immunohistochemistry analyses revealed that approximately 70% of DCT^+^ MSCs in the bulge/sHG niche of hair follicles also express SOX10 and C-KIT. Furthermore, C-KIT-positive cells were occasionally observed in the dermis, suggesting the potential of C-KIT to target MSCs (Sun et al., 2019; Infarinato et al., 2020) (Figure 1).

Skin melanocytes have a neural crest origin and can be indirectly derived from Schwann cell progenitors (SCPs) after peripheral nerve colonization (Adameyko et al., 2009; Kaucka et al., 2021) (Figure 1). SCPs are NCC-derived cells expressing hub genes, including *Serpine2*, *Itga4*, *Ednrb*, *Dlx1*, *Dlx2*, *Sox8*, *Sox10*, *Erbb3*, *Foxd3*, *Ets1*, *Plp1*, and *Tfap2* (Kastriti et al., 2022). At approximately E13, SOX10^+^ MITF^+^ cells were associated with nerves but were not in direct contact with them in mice. Furthermore, these cells express DCT, a key enzyme involved in melanin-mediated pigment synthesis. The myelin proteolipid protein (PLP)-CreERT2 mouse model was used for fluorescent fate mapping to explore the origin of nerve-associated melanoblasts (Adameyko et al., 2009). This model specifically labels SCPs and Schwann cells (SCs), enabling a detailed examination. A considerable proportion of postnatal skin melanocytes was derived from SCPs in mice. Moreover, SCPs situated along the elongating spinal nerves have been identified as a source of melanocytes. These pigmented cells differentiate later than melanocytes, which originate directly from NCCs. Schwann cell markers PLP1 and fatty acid binding protein 7 (FABP7) were substantially expressed in melanoblasts. However, the PLP-CreERT2 marker is not exclusive to SCPs. Additionally, this marker tags nerves and melanoblasts at various developmental stages in the skin. Thus, researchers used a glial-specific Dhh-Cre/R26R reporter mouse model to specifically label SCPs. However, there was no evidence of melanocytes originating from the SCPs. A recent study using the same Dhh-Cre driver line on a pure FVB/N background, along with a fluorescent Cre reporter, successfully labeled melanocytes, indicating that most skin melanocytes originate from SCPs (Colombo et al., 2012; Hari et al., 2012; Bonnamour et al., 2021).

There are several melanocyte precursor cells in the human interfollicular epidermis. C-KIT^-^CD90^+^ cells have neural crest characteristics and can differentiate into multiple lineages (Michalak-Micka et al., 2022). Similarly, dermal stem cells with neural-crest-like characteristics expressed the p75 neurotrophin receptor (NGFRp75), nestin, and octamer-binding transcription factor 4 (OCT4). However, they did not express melanocyte markers and could differentiate into melanocytes in human skin. Additionally, they acquired E-cadherin expression and lost NGFRp75 expression upon contact with epidermal keratinocytes (Li et al., 2010) (Figure 1). Even in the absence of appendages, the tail skin of mice retains a stable number of melanocytes, with a relatively low frequency of amelanotic and actively cycling differentiated melanocytes (Glover et al., 2015). This indicates that non-follicle-associated stem cells may contribute to interfollicular melanocytes.

### 2.1 The polarity of melanocytes

Melanocytes rely on various transcription factors and signaling systems for their development. MITF is vital for melanocyte survival, migration, proliferation, and differentiation. It also promotes the specification of NCCs into melanoblasts and influences their survival by affecting C-KIT expression (Opdecamp et al., 1997; Hou and Pavan, 2008). Melanoblasts derived from NCCs express MITF before the initial expression of DCT. These MITF-positive cells, derived from NCCs, are defined as melanocyte precursors based on the coexpression of markers, such as C-KIT and DCT (Opdecamp et al., 1997; Hou et al., 2000) (Figure 1). MITF and C-KIT signaling are required for tyrosinase expression in melanoblasts and influence gene expression during melanocyte development (Hou et al., 2000). Additionally, MITF comprises various isoforms with distinct 5′ exons and different promoters. However, melanocytes predominantly express the M-isoform (Hershey and Fisher, 2005). MITF contributes to melanocyte differentiation by modulating pigmentation genes, including *Tyr*, *Tyrp1*, *Dct*, *Pmel*, and *Mlan-a*. It also contributes to cell survival by regulating anti-apoptotic genes, including *Bcl2* and *Bcl2a1*. MITF further governs the cell cycle and melanocyte metabolism by regulating *Cdk2* and *Ppargc1a* (McGill et al., 2002). It forms protein complexes with the co-factors histone acetyltransferase p300 and cyclic adenosine monophosphate (cAMP) response element-binding (CREB) protein and recruits polybromo and Brahma-related gene 1 (BRG1)-associated factor (PBAF) chromatin remodeling complexes, along with SOX10, transcription factor ap-2 alpha (TFAP2A), and yin yang 1 (YY1), to melanocyte lineage enhancers to regulate pigment gene transcription (Hu et al., 2007; Qi et al., 2013; Laurette et al., 2015).

SRY-box transcription factor 2 (SOX2) and MITF determine the fate of SCP progenitors and melanocytes in the neural crest through cross-regulatory interactions (Figure 1). The direct regulation of MITF by SOX2 occurs through its inhibitory binding to the *Mitf* promoter during development. The gradual decrease in SOX2 levels facilitates the differentiation of melanocytes from progenitors of both the neural crest and SCP (Adameyko et al., 2012). The winged-helix forkhead transcription factor forkhead box d3 (FOXD3) plays a critical role in NCC specification. FOXD3 acts in a bimodal manner during NCC development, maintaining multipotency and determining cell fates by switching between “permissive” and “repressive” nucleosome and chromatin organization (Lukoseviciute et al., 2018). Distinct lineage tracing of each population indicated that melanocytes derived from NCCs and SCPs were restricted to distinct regions of the body. The epaxial and hypaxial domains contained only melanocytes derived from NCCs and SCPs, respectively. Populations of epaxial and hypaxial melanocytes originate from FOXD3-positive neural cells. However, epaxial melanocytes separate from neural progenitors in the dorsal neural tube and downregulate *Foxd3* expression, whereas hypaxial melanocytes lose *Foxd3* expression at later stages after separation from the nerve. Furthermore, timely downregulation of FOXD3 is necessary for proper differentiation of melanocyte populations derived from NCCs and SCP (Nitzan et al., 2013). β-catenin promotes the SCP-derived melanocyte specification through MITF repression of FOXD3 (Colombo et al., 2022). Then, FOXD3 interacts with PAX3 to inhibit its binding to *Mitf* (Thomas and Erickson, 2009). Thus, FOXD3 acts as a switch between SCPs and melanocytes (Figure 1).

Melanocyte specification relies on the correct delivery of signals from the intrinsic and extracellular environments, which must occur in a timely and spatially controlled manner. This process is primarily regulated by molecules such as WNT, stem cell factor (SCF), bone morphogenetic protein (BMP), and endothelin (EDN) 3. Double knockout mice lacking WNT1 and WNT3a exhibit an almost complete loss of melanocytes, indicating an impact on several neural crest (NC) derivatives (Ikeya et al., 1997). Similarly, knockout mice lacking EDN3 or its receptor endothelin receptor b (EDNRB) show a significant loss of melanocytes (Baynash et al., 1994). Combined treatment with BMP4 and EDN3 effectively induces the sequential generation of NC cells and melanocyte precursors from pluripotent stem cells (Mica et al., 2013). In addition, this process involves β-Catenin, a downstream signaling component of WNT. A complete loss of DCT and MITF expression was observed in mutant embryos of β-catenin at E10.5 and E12.5 (Hari et al., 2002). However, the effect of WNT/β-catenin on melanocyte fate decisions is temporary. Activation of β-catenin in NCCs before migration hinders the development of melanocytes, whereas it stimulates melanocyte generation in migrating NCCs (Hari et al., 2002; Hari et al., 2012). However, the initial determination of Schwann cell precursors remains unaltered in the absence of β-catenin. MITF can redirect the transcriptional function of β-catenin towards target MITF-specific promoters rather than the genes regulated by β-catenin/LEF1. Thus, β-catenin/TCF-dependent gene expression is diminished by the overexpression of MITF, whereas that dependent on MITF is augmented by the overexpression of β-catenin (Schepsky et al., 2006). The interplay between WNT and Frizzled receptors (FZD), coupled with their co-receptor low-density lipoprotein receptor-related protein (LRP), stabilizes cytosolic β-catenin. As a result, the transported cytosolic β-catenin enters the nucleus, where it engages and interacts with transcription factors LEF/TCF to assert regulatory dominion over MITF transcription (Steingrimsson et al., 2004; Perugorria et al., 2019).

RNA editing is a crucial process involving the conversion of adenosine into inosine (A to I). The enzyme A-to-I deaminase (ADAR) plays an important role in this process. Conditional NCC ADAR1 knockout leads to global depigmentation, owing to melanocyte apoptosis without immune cell recruitment. The expression of melanocytic markers (SOX10, DCT, and TYR) was downregulated, whereas that of interferon-stimulated genes (ISG signature), including *Cxcl10*, *Isg15*, *Ifit1*, *Ifit2*, *Rsad2*, and *Mx1*, was considerably upregulated in half of the mutants. This was attributed to abnormal interferon production mediated by melanoma differentiation-associated protein 5 (MDA5) at E18.5. In contrast, no disparity was observed between mutants and controls at E16.5, the phase during which melanoblasts commonly initiate their transformation into hair follicles. This indicates that RNA editing plays a crucial role in the survival of melanocytes from E18.5 onwards (Gacem et al., 2020). However, additional studies are required to establish whether alterations occur in the functionality of mutant melanoblasts.

### 2.2 The migration of melanocytes

Melanocyte migration is regulated by several signaling pathways and transcription factors. The invasion of the skin by melanoblasts occurs when they enter the dermis at E9.5 during embryonic development. Melanoblasts successfully surpassed the basement membrane (BM) and established their residence in the epidermis at approximately E12.5 and E13.5. The migration of melanoblasts into growing HFs began at E15.5. Melanocytes initially migrate to the upper region of the HF, called the hair bulge, which is the permanent portion and thereafter function as the MSCs population. They continue to migrate as the anagen stage progresses, reaching the most distal part of the HF, known as the hair bulb and thereafter function as melanocytes. Postnatally, the presence of melanocytes in the epidermis of the back skin ceases in mice, and they are exclusively found within HFs, functioning as cells responsible for melanin production (Nishimura et al., 2002; Nishimura et al., 2005; Mort et al., 2015). Imaging and mathematical modeling studies on murine melanoblasts have shown that the migration of melanoblasts in developing dermis at E11.5–E12.5 or epidermis at E13.5–E15.5 do not have directionality (Mort et al., 2016).

Highly mobile melanoblasts move across the dermis and epidermis during embryogenesis. These cells use myosin motors to extend the short and long pseudopodia, enabling navigation through the epidermal keratinocyte layer. The rates of actin assembly and pseudopod extension in melanoblasts are controlled by Rac Family small GTPase 1 (RAC1), a Rho-family small GTPase, with stabilization provided by the actin-bundling protein fascin (Li et al., 2011; Ma et al., 2013). Furthermore, RAC1 activation is facilitated by an integrin-linked kinase (ILK) gene. The deletion of RAC1 and ILK in melanoblasts causes defects in cell migration, cell cycle progression, and cytokinesis (Crawford et al., 2020). Cell division control protein 42 homolog (CDC42) is another crucial regulator of the migration and proliferation of melanoblasts. Its absence results in various impairments in melanoblast migration, including disruption of actin dynamics, contractile activity, and adhesion (Woodham et al., 2017). Melanoblasts migration to the epidermis is accompanied by a change in the surface phenotype from E-cadherin^low^ P-cadherin^high^ to E-cadherin^high^ P-cadherin^low^, which is reversed when migrating from the epidermis into hair follicles displaying the same patterns as surrounding cells in terms of expression of E- and P-cadherin (Nishimura et al., 1999). Furthermore, the efficiency of melanoblasts migration is related to precise coordination of integrin-mediated cell-extracellular matrix (ECM) adhesion (Haage et al., 2020). However, how different cadherins and integrins are regulated during melanoblasts migration still needs further investigation.

**FIGURE 2:**
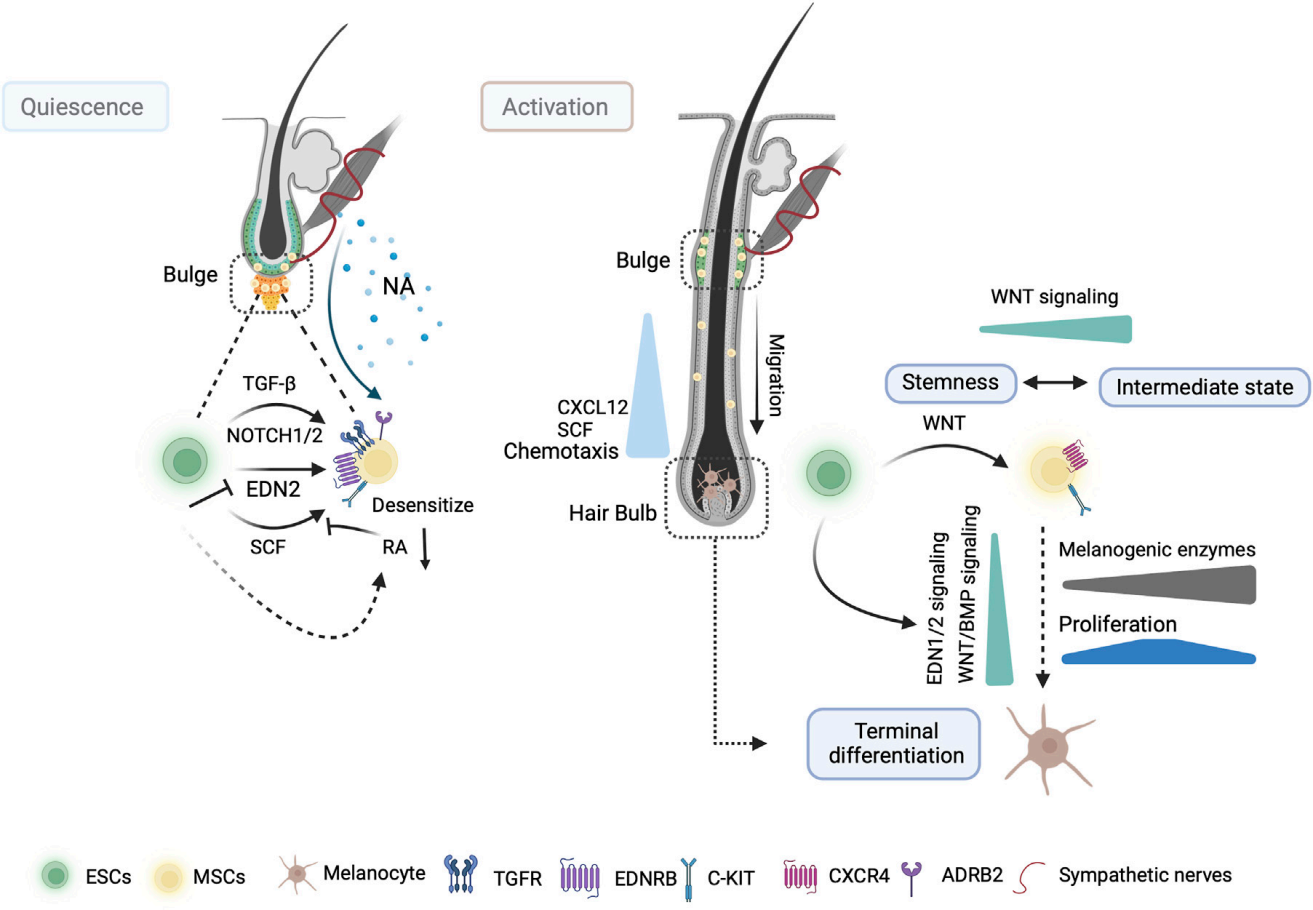
MSCs maintenance and activation in hair follicle stages. During telogen, niche ESC-derived TGF-β and Notch signaling synergistically maintain MSC stemness. Additionally, ESCs desensitize MSCs to differentiation signaling from SCF and EDN2 by downregulating retinoic acid (RA) levels in the niche and NFIB signaling, respectively. Sympathetic nerves terminate close to hair bulge MSCs and release noradrenaline (NA) to trigger MSCs depletion through ADRB2 receptor. Following the WNT-mediated activation of MSCs by niche ESCs, the BMP, WNT, and END1/2 pathways collaborate to trigger melanocyte proliferation and differentiation during anagen. Upon activation, SCF from the dermal papilla and CXCL12 of the outer hair root sheath direct the migration of MSCs towards the hair bulb. Some activated MSCs revert to a stem-like state upon migration into the WNT-negative bulge. This illustration was created using BioRender.com.

Colonization of the epidermis and HFs by melanocytes is an active process that requires functional C-KIT/SCF signaling. Forced expression of SCF in *K14-Scf* transgenic mice stimulates the proliferation, differentiation, and movement of melanoblasts during embryogenesis, along with MSCs during hair cycling. Consequently, an increase in the number of epidermal melanocytes and epidermal hyperpigmentation was observed. Additionally, robust SCF expression in the epidermis facilitates the migration of MSCs from the follicle to the epidermis on the dorsal skin of mice, particularly in response to ultraviolet radiation b (UVB) irradiation (Kunisada et al., 1998; Aoki et al., 2009; Yamada et al., 2013). Hair depigmentation was observed in *Scf* ^*flox/gfp*^ *::K14-Cre* mice, and upregulating the membrane-bound form of SCF in epidermal keratinocytes did not reverse it (Liao et al., 2017). Moreover, the offspring of mice administered blocking antibodies targeting the C-KIT during pregnancy had a white coat. This further confirmed the critical role of C-KIT/SCF signaling in facilitating melanoblast migration from the dermis to the epidermis and their subsequent localization within the follicles (Nishikawa et al., 1991). Recent studies have shown that SCF exhibits chemokinetic behavior rather than acting as a chemotactic factor (Jordan and Jackson, 2000). The movement of melanocytes into the HF throughout HF development and cycling is controlled by stromal cell-derived factor 1 (SDF-1), also known as C-X-C motif chemokine 12 (CXCL12), and its receptor, C-X-C chemokine receptor 4 (CXCR4). SDF-1 is expressed along the migration route followed by CXCR4-expressing cells in the outer root sheath of HFs. Additionally, CXCR4-positive cells in the HF bulge co-expressed nestin and LEX, which are markers of stem cells, along with SOX10, a marker of NCCs, and DCT, a marker of melanoblasts. During the normal positioning of melanoblasts in the skin at E13.5, the CXCR4 antagonists can lead to melanoblast concentrate in the epidermis without migration into HF. This suggests that SDF-1 promotes the migration of melanoblasts into HF (Figure 2). However, CXCR4 expression decreases considerably in differentiating melanoblasts and is enhanced during the anagen phase of the HF cycle (Belmadani et al., 2009). These findings indicate that external signals play a crucial role in melanoblasts/melanocytes movement. Moreover, it is imperative to adjust receptors associated with their surroundings and signaling alterations (Liao et al., 2017). However, the mechanism by which CXCR4 expression is regulated in melanocytes at different hair follicular stages remains unclear.

Melanocyte migration is not a one-way process. After wounding or UVB irradiation, MSCs can leave niches and generate epidermal melanocytes in the skin. The migration and differentiation of MSCs into melanocytes in the epidermis occur without proliferation and are dependent on the presence of the melanocortin 1 receptor (MC1R) (Figure 1). Furthermore, the number of MSCs directly migrating to the epidermis of the mouse back skin after wounding is lower in *Mc1r* mutant mice with nonfunctional MC1R than in littermate controls (Chou et al., 2013). In addition, the transgenic expression of *Dkk1*, an inhibitor of the WNT pathway, in melanocytes decreased their migration to the epidermis in the dorsal area of mouse wound scars. However, activating WNT signaling by genetically stabilizing β-catenin in melanocytes enhances the production of epidermal melanocytes. Impairing the secretion of WNT ligands, which activate the WNT pathway in basal epidermal keratinocytes, hinders WNT signal activation in epidermal melanocytes. However, the suppression of WNT ligand release by melanocytes does not affect their WNT stimulation, indicating that the paracrine communication of WNT from epidermal keratinocytes plays a vital role in the migration of MSCs towards the epidermis (Sun et al., 2018). In contrast, overexpression of EDN1 within epithelial cells can compensate for the impaired regeneration of epidermal melanocytes triggered by MC1R loss. This compensation occurs through synergistic effects with the WNT pathway, which enhances MSC proliferation and migration. Loss of EDNRB in MSCs compromises their migration to the epidermis after wounding, as demonstrated through EDNRB knockout, specifically in the melanocyte lineage in mice (Takeo et al., 2016). SCP-derived melanoblasts require EDNRB and WNT5a for expansion and EDNRB for migration (Adameyko et al., 2012). These results show that MSCs require both extrinsic and intrinsic cues to drive their migration. These findings further demonstrate that MSC migration is not static and is directed by environmental signals.

### 2.3 MSCs grow and rest in hair

HF growth undergoes numerous cycles, including different phases: anagen (growth phase), catagen (regression phase), and telogen (resting phase). These phases are driven by the proliferation and differentiation of epithelial stem cells (ESCs) that express keratin 15 (K15) in the bulge area and sHG (Greco et al., 2009; Zhang et al., 2009). MSC differentiation and melanogenesis are coupled with distinct phases of the hair cycle (Slominski and Paus, 1993). MSCs can produce progeny that undergo rapid proliferation during the early anagen phase. These progeny subsequently mature into melanocytes that synthesize and transmit melanin to differentiating hair cells in the hair bulb, giving the hair a dark color (Infarinato et al., 2020). MSCs and ESCs are activated in coordination at the initiation of the new anagen phase, and synchronized activity continues throughout the hair cycle. Activation of WNT signaling in K15^+^ ESCs can activate and enhance the proliferation and differentiation of MSCs (Figure 2). This is partially achieved through the secretion of EDN1, a potent mitogen in melanocytes. MSCs express EDNRB, which binds to EDN 1, 2, and 3 to transduce EDN ligand signals at the onset of anagen (Sakurai et al., 1990; Saldana-Caboverde and Kos, 2010; Rabbani et al., 2011; Takeo et al., 2016). Some WNT-activated MSCs can differentiate into an intermediate state and revert to a stem-like state upon migration into a WNT-negative bulge area (Sun et al., 2023) (Figure 2). Moreover, MSCs undergo premature differentiation when the WNT/FZD pathway is activated. However, their differentiation into hair bulb melanocytes is impaired in the absence of β-catenin (Rabbani et al., 2011). Additionally, the coordination of epithelial-MSCs behavior is regulated by the transcription factor Nuclear Factor I B (NFIB), which negatively regulates EDN2 and inhibits MSC differentiation in a C-KIT-dependent manner in ESCs (Chang et al., 2013) (Figure 2). SCF acts as a dermal papillary signal that promotes MSC migration and differentiation. Its overexpression in the hair follicle epithelium increased the proliferative activity of melanocytes (Figure 2). When a C-KIT antibody was injected during the catagen/telogen stage in anagen hair follicles, it only affected the proliferation and differentiation of lineage-committed cells. However, undifferentiated MSCs remained elevated in the hair follicles of *Nfib* ^fl/fl^-*K15*-cre mice, suggesting that MSCs respond to SCF differently depending on the stage of development and the environment in which they reside (Botchkareva et al., 2001). During onset of catagen development, the maximal expression of transforming growth factor-β (TGF-β) by bulge outer root sheath was observed (Foitzik et al., 2000). This may lead to the apoptosis of melanocytes in catagen (Sharov et al., 2005; Nishimura et al., 2010).

The bulge must sustain a sufficient number of MSCs for subsequent hair growth cycles to ensure sustainability (Infarinato et al., 2020). Niche ESCs play a crucial role in the telogen phase (Nishimura et al., 2010). TGF-β signaling from ESCs temporarily halts the cell cycle and supports melanocyte immaturity by reducing MITF levels (Figure 2). During the hair cycle, the activation of TGF-β signaling occurs in MSCs as they transition into quiescence state and requires B-cell lymphoma 2 (BCL-2) to antagonize the pro-apoptotic effect of TGF-β. In addition, loss of TGF-β type II receptor (TGFbRII) within the melanocyte lineage leads to the incomplete preservation of MSC immaturity (Nishimura et al., 2010). Collagen XVII (COL17A1), highly expressed in ESCs, maintains TGF-β signaling by mediating anchorage (Nishimura et al., 2005; Nishimura et al., 2010; Tanimura et al., 2011). Moreover, MSCs within their niche rely on BRAF and CRAF for self-maintenance. These two RAF proteins are functionally redundant and compensate for each other. The RAF/extracellular signal-regulated kinase (ERK) pathway regulates the cell cycle, and the absence of RAF proteins in knockout mice affects the entry of MSCs into S phase (Valluet et al., 2012). Furthermore, the Notch signaling pathway determines the fate of MSCs and melanoblasts. In skin organ cultures, the inhibition of Notch signaling led to the apoptosis of MSCs and melanoblasts (Figure 2). Conversely, overexpression of *Hes1*, specifically in MSCs and melanoblasts, protects melanoblasts from apoptosis by preventing its initiation (Moriyama et al., 2006; Kumano et al., 2008). Although C-KIT signaling plays a vital role in the migration, survival, and differentiation of MSCs, its presence is not required for the self-maintenance of MSCs within their niche. However, ESCs regulate retinoid metabolism, which leads to the downregulation of retinoic acid levels, to desensitize MSCs to differentiation signaling from the C-KIT ligand (Lu et al., 2020) (Figure 2). These results show that signaling from ESCs contributes to the preservation of their regenerative potential by maintaining a self-renewal niche for MSCs.

The transcriptional features of MSCs as they progress through the different stages of their lineage are complex. MSCs and their progeny universally express certain pan-lineage genes such as *Sox10*. They displayed selective upregulation of gene expression, including that of *Pax3*, *Sbno2*, and *Bcl2* during the transition from the quiescent to the activated stage, accompanied by high expression of proliferation-related genes (i.e., *Mki67* and *Ccna2*) (Figure 1). In contrast, the transcripts involved in pigment production, such as melanin production enzymes (TYR and TYRP1) and melanosome-related proteins (MART1 and Oculocutaneous albinism type 2 (OCA2)), were elevated during the transition from proliferation to maturity (Figure 1). Mature melanocytes exhibited considerably lower transcript levels of key cell cycle genes than those in the activated stage. Additionally, they were enriched in C-KIT/SCF, WNT signaling (LEF1), and BMP signaling (Infarinato et al., 2020) (Figure 2). However, unlike those in the secondary hair germ (sHG) (CD34^−^ area), MSCs within the bulge (CD34^+^ area) did not activate WNT signaling at anagen onset. Furthermore, they proliferated even after induction by the WNT ligand (Rabbani et al., 2011). Fluorescence-activated cell sorting recently demonstrated that CD34^+^ and the CD34^-^ MSCs are functionally distinct. The expression of key melanocyte differentiation genes, including *Mitf*, *Tyr*, *Tyrp1*, *Pmel*, *Pax3*, *Mc1r*, *Erbb3*, *Sox10*, *Melan-A*, and *Slc45a2*, was elevated in CD34^-^ MSCs. In contrast, CD34^+^ MSCs displayed a gene expression profile more consistent with a neural crest stem cell, with higher *Nr2f2, Nr2f1, Ngfr (p75)*, *Twist1*, *Twist2*, *Snai1*, *Sox9*, *EdnrA*, *Gli1*, *Bmp2*, *Bmp4*, and *Bmp7* levels observed (Joshi et al., 2019). These observations suggest that there may be intrinsic heterogeneity among follicular MSCs with differential responsiveness to WNT ligands and SCF. However, additional research is required to fully evaluate this possibility.

### 2.4 Melanin synthesis

Melanocytes mainly produce two types of melanin: eumelanin and pheomelanin. Eumelanin is dark brown and is the primary pigment for protection against ultraviolet radiation (UVR). In contrast, pheomelanin is reddish-yellow and is more susceptible to UVR-induced damage. Melanocytes, which are cells responsible melanin production, generate this pigment within cellular structures known as melanosomes. Melanosomes can be categorized into four stages, ranging from early and unpigmented (stages I–II) to late and pigmented (stages III–IV). These stages can be distinguished based on their morphology. Stage I melanosomes exhibit non-pigmented vacuoles, whereas stage II melanosomes exhibit internal striations. Melanin gradually accumulates on fibrils during stage III, eventually leading to the fully melanized stage IV (Tian et al., 2021). Mature melanosomes are ultimately transferred to epidermal keratinocytes. They then aggregate above the nuclei of keratinocytes that face the sun (Park et al., 2009).

The enzymatic machinery and structural components of melanosomes must be appropriately assembled into newly formed melanosomes. Melanosome formation begins with fiber formation by melanosome structural proteins, such as PMEL17. MART1 binds to this structural protein to facilitate its expression, stability, and trafficking (Tian et al., 2021). The formation of PMEL17 amyloid fibers is affected by pH levels and is most suitable for mildly acidic pH levels (4.5–5.5) (Pfefferkorn et al., 2010). V-ATPase, an H^+^ pump, is expressed in stage I premelanosomes, creating an acidic environment (Tabata et al., 2008). Melanogenesis begins at stage III, when TYR, TYRPs, ATPase copper transporting alpha (ATP7A), OCA2, SLC45A2, and two pore segment channel 2 (TPC2) are located from the trans-Golgi network to melanosomes. ATP7A functions as a Cu^2+^ pump, providing Cu^2+^ as a TYR cofactor. OCA2, SLC45A2, and TPC2 are membrane transport proteins that are essential for converting acidic pH to neutral pH (Le et al., 2020; Wiriyasermkul et al., 2020). Enzymes, such as TYR, TYRP1, and DCT, are required to initiate the oxidation of tyrosine to L-DOPA during melanin production in fully striated melanosomes. Subsequently, L-DOPA is transformed into DOPAquinone, which serves as a precursor of eumelanin and pheomelanin (Raposo and Marks, 2007; Centeno et al., 2023). TYR degradation is regulated by nicotinamide nucleotide transhydrogenase (NNT), an enzyme involved in the mitochondrial redox-regulating pathway, through a ubiquitin-proteasome mechanism (Allouche et al., 2021). A CRISPR-Cas9 genetic screen recently uncovered 135 melanin-promoting genes that had not been previously identified. A notable decrease in melanin production was observed upon the deletion of these genes. The identified melanin-promoting genes play crucial roles in various biological pathways, including transcriptional regulation, RNA processing, and endosomal transport. Depletion of the transcript factor *KLF* downregulated TYR expression. Furthermore, the endosomal trafficking protein COMM Domain Containing 3 (COMMD3) is required for neutral melanosomal pH maintenance (Bajpai et al., 2023).

### 2.5 Melanin transfer to keratinocytes

**FIGURE 3:**
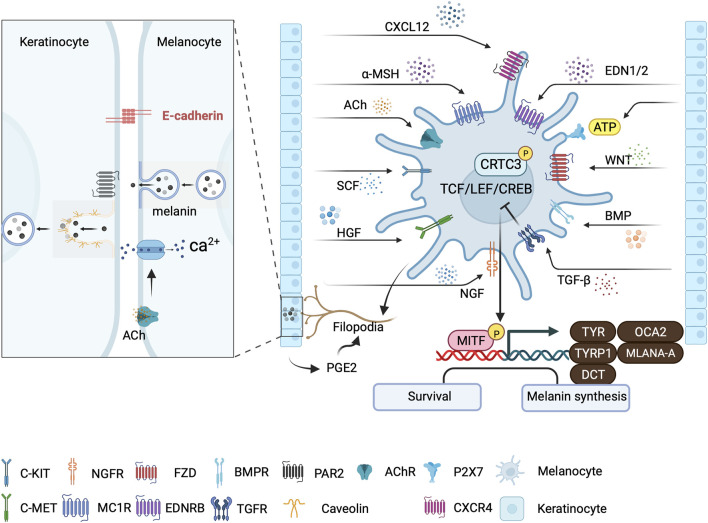
Major signaling pathways involved in skin pigmentation and melanin transfer. Keratinocytes regulate melanogenesis through various paracrine signaling pathways that converge at the CRTC3/CREB/MITF axis. Signaling from neighboring keratinocyte-secreted factors, including acetylcholine (ACh), can trigger localized Ca^2+^ transients that initiate melanin transfer within melanocyte dendrites. PGE2 from keratinocytes stimulate melanocyte filopodia formation. Melanocytes: Keratinocyte-contact-dependent melanin transfer is mediated by E-cadherin and Cav1/caveolae, whereas melanin secreted by melanocytes is phagocytosed by keratinocytes in a PAR-2 dependent manner. This illustration was created using BioRender.com.

The skin is the primary barrier to the external environment and relies on melanin for photoprotection (Singh et al., 2017). Melanin transfer from melanocytes to keratinocytes is influenced by calcium flux regulation. UVR also elevates intra-melanocyte Ca^2+^ levels by activating calcium release-activated calcium Modulator 1 (ORAI1) ion channels that are calcium selective. This leads to upregulation of the expression of filopodia-associated proteins, including E-Cadherin, CDC42, vasodilator-stimulated phosphoprotein (VASP), and β-catenin (Figure 3). The inhibition of E-cadherin expression and knockdown of transient receptor potential cation channel subfamily M member 1 (TRPM1) reduces the transfer of melanosomes from melanocytes to keratinocytes following exposure to ultraviolet radiation a (UVA) or UVB radiation (Hu et al., 2017; Singh et al., 2017). EDN and acetylcholine from keratinocytes trigger localized dendritic Ca^2+^ transients within melanocyte dendrites *in vitro* (Belote and Simon, 2020) (Figure 3). The coordination of melanocyte-keratinocyte communication and contact by caveolae is also crucial for melanosome transfer to keratinocytes. UVR and keratinocyte-released factors lead to the preferential location of caveolae at the melanocyte-keratinocyte interface (Figure 3). Cav1/caveolae downregulation induces pigment production in melanocytes by increasing cAMP production, whereas upregulation favors changes in cell morphology and promotes contact with keratinocytes, both of which lead to melanin transfer and skin pigmentation. These findings demonstrate that melanocytes effectively respond to external signals from keratinocytes by utilizing the signaling and mechanistic mechanisms of the caveolae (Domingues et al., 2020).

Pigmentation occurs when melanin is synthesized within lysosome-like structures known as melanosomes by melanocytes and then delivered to keratinocytes (Raposo and Marks, 2007). There are four possible models of melanosome transfer from melanocytes to keratinocytes mainly based on *in vitro* experiments (Wu and Hammer, 2014; Tadokoro and Takahashi, 2017). In the cytophagocytosis model, keratinocytes can phagocytize entire constituents of melanocytes (e.g., dendrites or filopodia) as a means of internalizing melanin. In the fusion model, melanosomes move along filopodia and form a channel into the keratinocyte. It is believed that filopodia fuse with the keratinocyte membrane during melanosome transfer. In the vesicle transfer model, membrane vesicles containing either individual or groups of melanosomes are released from the melanocyte and then captured and phagocytized by keratinocytes. In the exocytosis model, melanin units without their melanosomal membranes (termed melanocores) are released into the extracellular space between the melanocyte and keratinocyte. Subsequently, the melanocores are internalized by keratinocytes through phagocytosis, which relies on the activation of protease-activated receptor-2 (PAR-2) (Wu and Hammer, 2014; Tadokoro and Takahashi, 2017; Moreiras et al., 2022). Following the transfer of melanin to keratinocytes, the cytoplasmic intermediate protein chains of dynein (Dync1i1) link the entire motor complex and the p150^Glued^ (DCTN1) subunit of the dynactin complex, which regulates the localization of perinuclear melanin (Byers et al., 2003; Byers et al., 2007). The calcium-dependent G protein-coupled and Akt signaling pathways upregulate the expression of DCTN1 in keratinocytes after exposure to UVA radiation. This process is facilitated by opsin3 (OPN3) (Lan et al., 2023). Melanocyte uptake induces melanin accumulation inside keratinocytes within hybrid endocytic compartments that exhibit low acidity and limited degradation capacity. These distinct endosomes enable melanin to persist in keratinocytes for an extended duration by evading lysosomal degradation (Correia et al., 2018).

### 2.6 Ultraviolet-induced pigmentation

Skin pigmentation, a crucial defense process against UV light, involves key cellular players in the epidermis, namely, melanocytes and keratinocytes. The close interconnections between these cellular types play a vital role in the regulation of skin pigmentation. Although the regulation of melanin biosynthesis involves various signaling pathways and factors, its pivotal role in UVB-induced MITF expression and melanogenesis can be attributed to cAMP and CREB (Yoo et al., 2021). UVR induces melanocyte-stimulating hormone (α-MSH) secretion in keratinocytes in a p53-dependent manner (Cui et al., 2007). The binding of α-MSH to receptor MC1R on melanocytes triggers the signaling pathway, which begins with Gαs activation of adenylate cyclase (Figure 3). This increases cAMP levels and phosphorylation of CREB transcription factor family members through PKA signaling (Cui et al., 2007). CREB transcriptionally activates MITF, which induces TYRP1, DCT, and TYR, subsequently driving melanosome maturation and increasing eumelanin production (Iozumi et al., 1993; Levy et al., 2006; Paterson et al., 2015; Tian et al., 2021) (Figure 3). The elevation in cAMP levels also significantly decreases the levels of CLEC12B, a C-type lectin receptor that inhibits CREB protein degradation (Sormani et al., 2022). Additionally, cAMP-regulated transcription co-activators 1–3 (CRTC1-3) are sensors and key regulators of melanogenesis that act as effectors of cAMP signaling (Yoo et al., 2021). Salt-inducible kinases (SIKs) initially retain CRTCs in the cytoplasm through phosphorylation at the 14-3-3 binding sites. Elevated cAMP levels promote protein kinase A (PKA)-mediated phosphorylation, which inhibits SIKs. Then, nuclear migration occurs following CRTC dephosphorylation, leading to recruitment to the binding sites of CREB. This recruitment is essential for activating CREB complex-mediated transcription, which is necessary for complete activation of CREB-mediated transcription (Altarejos and Montminy, 2011). In contrast to the nonspecific stimulation of all CRTCs caused by high cAMP levels, CRTC3 is synergistically activated by ERK1/2 and cAMP during low cAMP signaling in melanocytes. Melanocyte mutants with CRTC3 exhibit flawed maturation of melanosomes, owing to the reduced expression of OCA2, an essential pigment regulator (Ostojic et al., 2021) (Figure 3).

Paracrine signals from keratinocytes modulate melanogenesis. Basic fibroblast radiation factor (bFGF) expression is upregulated in keratinocytes and promotes melanocyte proliferation and melanin synthesis (Halaban et al., 1988). It binds to its receptor on melanocytes to enhance PAX3 expression (Dong et al., 2012). UV irradiation also upregulates the expression of keratinocyte-derived nerve growth factor (NGF), which is chemotactic in melanocytes and induces their dendricity (Yaar et al., 1991). NGF helps preserve UV-induced depletion of cutaneous melanocytes by increasing the expression of the anti-apoptotic BCL-2 protein (Stefanato et al., 2003). Hepatocyte growth factor (HGF), mainly produced by keratinocytes and fibroblasts, is the exclusive ligand of cellular mesenchymal epithelial transition (c-MET) factor, a membrane-bound receptor with kinase activity (Upadhyay et al., 2021). HGF/c-MET signaling activates mitogen-activated protein kinases (MAPKs) and phosphoinositide 3-kinases (PI3K)/protein kinase B (AKT) signaling to modulate CREB activity. It further affects melanocyte proliferation, motility, and survival. UV-induced secretion of interleukin (IL)-1α by keratinocytes also upregulates HGF synthesis by fibroblasts (Mildner et al., 2007; Czyz, 2018). After UVB radiation, adenosine 50-triphosphate (ATP) release increases in keratinocytes. Thus, ATP can act as an extracellular signaling molecule by activating cell surface P2X7 receptors to promote melanin production in melanocytes. Although ATP increases intracellular Ca^2+^, p-CREB, and MITF levels in melanocytes, an inhibitor of protein kinase C (PKC) abrogates this effect. This suggests that the Ca^2+^ influx/PKC/p-CREB/MITF axis is involved in ATP-induced melanogenesis (Khakh and North, 2012; Lee et al., 2019). Filopodial-associated E-Cadherin, VASP, CDC42, and β-catenin, whose expression was upregulated by UVR/UVA and Ca^2+^ in melanocytes, are required for melanin transfer (Singh et al., 2017) (Figure 3). Exosomes, soluble factors released by keratinocytes, carry membrane proteins and cytosolic components modulated by UVB. In addition, they could play a role in the regulation of melanogenesis (Lo Cicero et al., 2015). Low-dose UVB irradiation induces the expression of the gene *Col2a1*, encoding ECM. Collagen type II alpha 1 (COL2A1) promotes melanogenesis through activation of the MAPK pathway (Li et al., 2021).

**FIGURE 4:**
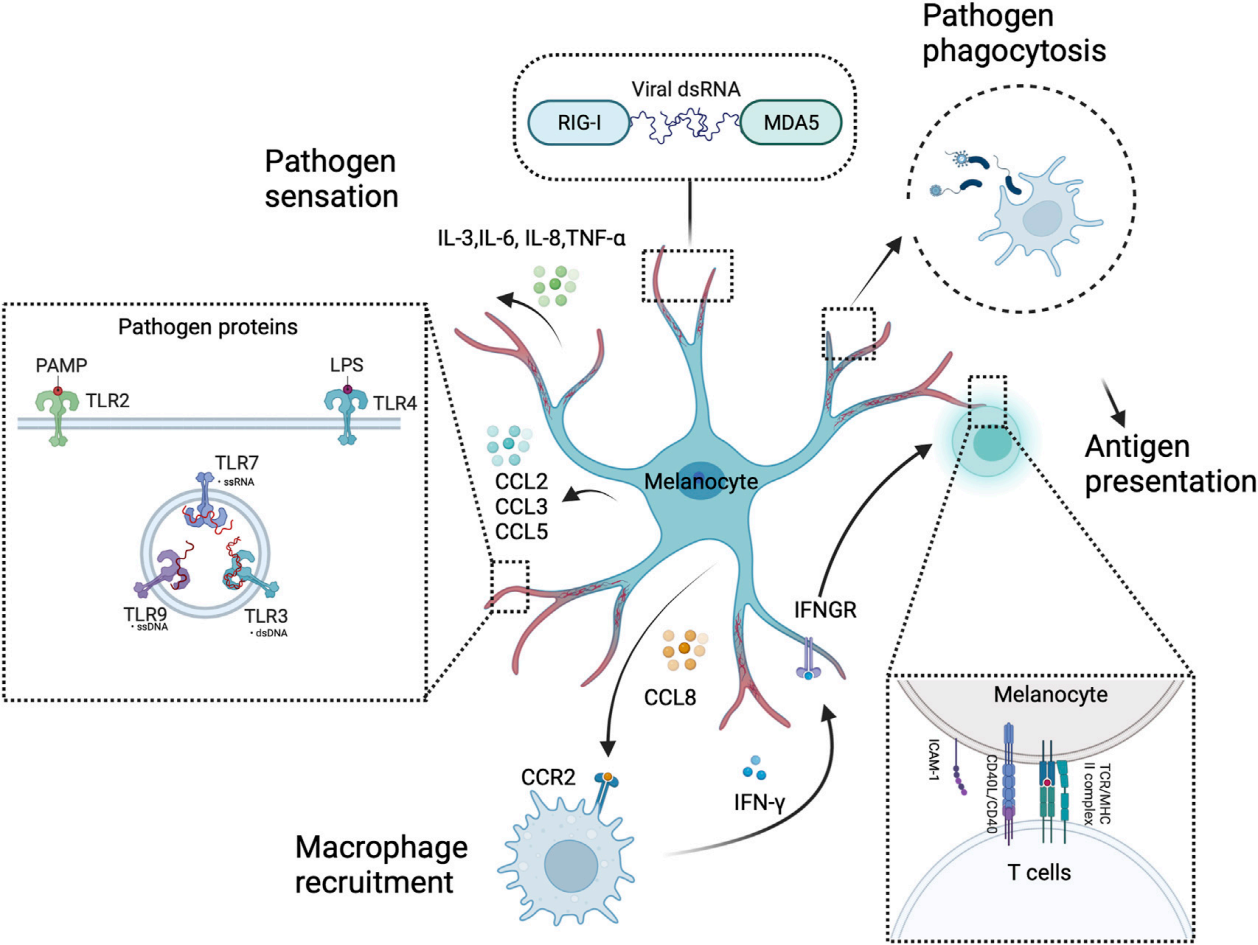
Melanocytes as sentinel immune cells in the skin. Melanocytes sense and respond to pathogens through intracellular or extracellular pattern recognition receptors, such as RIG-I, MDA5, and TLRs. These receptors activate melanocytes. Then, innate immune molecules (e.g., chemokines and cytokines) are produced to alert professional immune cells (e.g., macrophages) in a concerted effort to thwart off pathogens. Furthermore, melanocytes have a pathogen phagocytosis ability and present antigens to T cells. This illustration was created using BioRender.com.

Inflammatory responses are also involved in UVB-induced melanocyte proliferation and skin pigmentation. UVB radiation stimulates the release of the C-C chemokine receptor type 2 (CCR2) ligand C-C Motif Chemokine Ligand (CCL) 8 by melanocytes, which attracts CCR2^+^ macrophages to the skin. The recruited macrophages produce Interferon (IFN), which promotes melanocyte proliferation and migration (Zaidi et al., 2011) (Figure 4). However, Ly6c^low^MHCII^hi^ macrophages primarily promote melanocyte reactivation, which is partly IL-17 dependent and CCR2 independent (Handoko et al., 2013). UV exposure increases keratinocyte synthesis of IL-1α, IL-6, and tumor necrosis factor (TNF)-α (Kock et al., 1990; Chung et al., 1996), which also inhibit melanocyte proliferation and melanogenesis (Swope et al., 1991). UVR induces the release of prostaglandin E(2) [PGE(2)] by keratinocytes, thereby stimulating filopodia formation in melanocytes. PGE(2) also stimulates EP4 receptor signaling in melanocytes, resulting in increased tyrosinase activity and proliferation (Starner et al., 2010) (Figure 3).

### 2.7 Innervation and pigmentation

An earlier study found that intraepidermal free nerve endings (IEFNs) were in contact with melanocytes. The ultrastructural features of the chemical synapses can be observed in these contacts. IEFNs show enlarged endings that form boutons closely adjacent to melanocyte cell bodies, resulting in membrane-to-membrane appositions. Furthermore, both neurites and melanocytes exhibited membrane thickening in proximity to vesicles that aggregated in nerve fibers, indicating possible efferent communication from IEFNs to melanocytes. Additionally, certain fibers complete their course by being enveloped within melanocyte cytoplasmic invaginations (Hara et al., 1996). A higher occurrence of contact between sensory neuron terminals and melanocytes was observed within the area of senile lentigo (SL) than in the encompassing region. Repulsive guidance molecule B (RGMB) secreted by neurons promotes melanogenesis, and the survival of melanocytes is higher in senile lentigo (SL) than in the healthy surrounding skin (Chow et al., 2022). Similarly, sympathetic nerves terminate close to hair bulge MSCs and release noradrenaline to trigger MSC depletion through adrenoceptor beta 2 (ADRB2) receptor (Zhang et al., 2020) (Figure 2). In addition, stimulation with the calcitonin gene-related peptide (CGRP) induced melanocyte proliferation and increased intracellular cAMP accumulation (Toyoda et al., 1999). However, the prevalence of CGRP-expressing IEFNs in the central depigmented region, which lacks melanocytes, exceeds that in unaffected vitiligo skin (Lazarova et al., 2000). These results suggest that the cutaneous nervous system disrupts melanocyte regulation under pathological skin conditions.

### 2.8 Melanocytes as immune cells in the skin

Melanocytes are not only responsible for melanin production. They also actively contribute to the functioning of the cutaneous immune system. The large surface area, dendritic structure, and strategic location of the superficial layers of melanocytes provide the structural foundation and potential to serve as skin sentinel cells (Plonka et al., 2009). Human melanocytes express functional toll-like receptors (TLRs), including TLRs 2, 3, 4, 6, 7, and 9. *In vitro* stimulation of TLR2 and 4 by Poly I:C and Lipopolysaccharide (LPS) induced IL-8 and IL-6 production in melanocytes, respectively. Furthermore, TLRs 2, 3, 4, 6, and 9 stimulation upregulated the expression of chemokines involved in leukocyte recruitment (CCL2, CCL3, and CCL5), which was not the case with TLR7 stimulation (Yu et al., 2009; Yu et al., 2021). Melanocytes also express other pattern recognition receptors (PRRs), including retinoic acid-inducible gene (RIG)-I-like receptors (RLRs) and MDA5, to detect and respond to pathogens. These receptors will initiate signaling pathways that promote the expression of type-I interferons (IFN-α/β) (Besch et al., 2009; Wang et al., 2015). Melanocytes have phagocytic abilities and most express MHC class I molecules. They also express MHC class II molecules through IFN-γ stimulation *in vitro* (Le Poole et al., 1993; Hedley et al., 1998). IFN-γ stimulation promotes CD40 expression by melanocytes. It further promotes the expression of the co-stimulating molecule intercellular adhesion molecule 1 (ICAM-1) following CD40 ligation, indicating the melanocyte ability to bind antigens and activate T cells (Lu et al., 2002; Polisetti et al., 2021). Melanocytes can also produce IL-3, IL-6, and TNF-α to trigger the maturation of plasmacytoid dendritic cells (pDCs) (Halasi et al., 2023). However, their immunological capacity remains largely unexplored, and most empirical observations have been derived from *in vitro* studies. Therefore, further investigations are required to establish a holistic understanding of the immunological function of melanocytes (Koike and Yamasaki, 2020) (Figure 4).

## 3 Discussion

This review elucidates the function, migration, and regulation of melanocytes in the skin. The contributions of melanocytes to cutaneous homeostasis, as well as the reliance of their migration on environmental signals have been elucidated in existing literature. Furthermore, the mechanistic mechanisms of the caveolae have been linked to melanocyte responses to external signals. Although technological advances have gradually revealed the function and regulation of melanocytes over the years, there remain many unknown aspects of their biology, owing to the complexity of their regulatory mechanism. Whether melanocytes can be derived from nerve cells, have nerve-like functions, can regulate keratinocyte function in addition to delivering melanin to keratinocytes, or have “autonomy” warrants further investigation. Furthermore, the mechanisms underlying the regulation of melanocytes by nerves in the skin remain unclear. Similarly, the mechanisms underlying the balance of different signaling pathways to enable melanocyte migration and function warrant further study. Therefore, a comprehensive understanding of melanocyte biology requires new research modalities. The role of melanocytes in the skin will become more prominent with further research, providing new perspectives on the pathogenesis and treatment of skin diseases.
